# Impact of the 24-hour time target policy for emergency departments in South Korea: a mixed method study in a single medical center

**DOI:** 10.1186/s12913-022-08861-y

**Published:** 2022-12-12

**Authors:** Sookyung Park, Hansol Chang, Weon Jung, Se Uk Lee, Sung Yeon Hwang, Hee Yoon, Won Chul Cha, Tae Gun Shin, Min Seob Sim, Ik Joon Jo, Taerim Kim

**Affiliations:** 1 School of Nursing, University of Virginia, 225 Jeanette Lancaster Way, Charlottesville, VA 22903-3388 USA; 2 Department of Emergency Medicine, Samsung Medical Center, Sungkyunkwan University School of Medicine, 115 Irwon-ro Gangnam-gu, Seoul, 06355 South Korea; 3 Department of Digital Health, Samsung Advanced Institute for Health Science & Technology (SAIHST), Sungkyunkwan University, 115 Irwon-ro Gangnam-gu, Seoul, 06355 South Korea; 4 Digital Innovation Center, Samsung Medical Center, 81 Irwon-ro Gangnam-gu, Seoul, 06351 South Korea

**Keywords:** Emergency departments, Length of stay, Health policy, Targets, Mixed methods

## Abstract

**Background:**

In South Korea, after the spread of the Middle East Respiratory Syndrome epidemic was aggravated by long stays in crowded emergency departments (EDs), a 24-hour target policy for EDs was introduced to prevent crowding and reduce patients' length of stay (LOS). The policy requires at least 95% of all patients to be admitted, discharged or transferred from an ED within 24 hours of arrival. This study analyzes the effects of the 24-hour target policy on ED LOS and compliance rates and describes the consequences of the policy.

**Methods:**

A mixed-methods approach was applied to a retrospective observational study of ED visits combined with a survey of medical professionals. The primary measure was ED LOS, and the secondary measure was policy compliance rate which refers to the proportion of patient visits with a LOS shorter than 24 hours. Patient flow, quality of care, patient safety, staff workload, and staff satisfaction were also investigated through surveys. Mann–Whitney U and χ2 tests were used to compare variables before and after the introduction of the policy.

**Results:**

The median ED LOS increased from 3.9 hours (interquartile range [IQR] = 2.1–7.6) to 4.5 hours (IQR = 2.5–8.5) after the policy was introduced. This was likely influenced by the average monthly number of patients, which greatly increased from 4819 (SD = 340) to 5870 (SD = 462) during the same period. The proportion of patients with ED LOS greater than 24 hours remained below5% only after 6 months of policy implementation, but the number of patients whose disposition was decided at 23 hours increased by 4.84 times. Survey results suggested that patient flow and quality of care improved slightly, while the workload of medical staff worsened.

**Conclusions:**

After implementing the 24-hour target policy, the proportion of patients whose ED LOS exceeded 24 hours decreased, even though the median ED LOS increased. However, the unintended consequences of the policy were observed such as increased medical professional workload and abrupt expulsion of patients before 24 hours.

**Supplementary Information:**

The online version contains supplementary material available at 10.1186/s12913-022-08861-y.

## Introduction

Increased length of stay (LOS) in crowded emergency departments (EDs) is a global problem [1, 2] that is associated with reduced quality of care [3–6]. A variety of techniques, such as team-based triage, fast-tracking, laboratory analysis in EDs, and nurse-requested X-ray imaging have been suggested [7]. Time targeted policies have also been proposed and implemented in several other countries, including the UK, Australia, New Zealand, and Canada [7–17].

In response to the Middle East Respiratory Syndrome (MERS) epidemic of 2015, South Korea introduced a “24-hour target policy” for EDs to prevent crowding and reduce the average LOS [18]. At the time of the South Korean MERS pandemic, among a total of 186 patients, 82 people were infected in one crowded ED due to a super-spreading event from one patient [19, 20]. This has led to a social consensus that ED crowding should be addressed. As a result, the 24-hour target policy was introduced in December 2017.

The 24-hour target policy requires at least 95% of all patients to be admitted, discharged, or transferred from the ED within 24 hours of arrival. Although there is a crowding disparity between EDs in Korea, mean proportion of patients who stayed in the ED for more than 24 hours reached 10% in crowded EDs [21, 22]. This characteristic crowding of Korean EDs led to a policy target time of 24 hours, which is longer than that used in other countries.

Setting targets might increase organizational performance; however, target-driven care risks distorting clinical priorities [23]. In previous studies, time targets in EDs have yielded controversial results, with both positive and negative consequences beyond their intended effects [7, 14–16, 24]. The effects of the South Korean policy on patients and medical professionals have yet to be studied and, given the longer target time of 24 hours compared to policies implemented in other countries, differences in impacts can be expected.

This study aimed to identify the impact of the 24-hour target policy in Korea on patients and medical professionals. This study used a mixed-methods approach to study the impact of the policy on LOS in EDs, policy compliance rates, and other consequences for patients and medical professionals.

## Methods

### Overall approach

The study is a retrospective observational study using a mixed-methods design to analyze ED visits and a survey of ED medical professional experiences. This study was conducted at a tertiary referral hospital in South Korea.

This study was approved by the institutional review board (IRB) of Samsung Medical Center. The need for informed consent was waived due to the retrospective, observational, and anonymous nature of the study by the institutional review board (IRB) of Samsung Medical Center. (IRB No. 2021–08-172). The survey of ED medical professionals was approved separately with informed consent (IRB No. 2021–08-173) of Samsung Medical Center.

### Participants and data sources

This retrospective study was conducted in the ED of a tertiary metropolitan hospital with approximately 1960 inpatient beds and approximately 80,000 ED visits per year. This study included ED visits from February 1, 2016, to June 31, 2019. The plan for implementing the 24-hour target policy was announced on July 10, 2017, and the policy was implemented on December 3, 2017 [18]. We classified the research period into three parts: pre-policy (February 2016 to June 2017), adjustment period (July 2017 to January 2018), and post-policy (February 2018 to June 2019). We compared outcome measures between the pre-policy and post-policy periods to evaluate the impact of the policy.

Survey responses were collected from 22 doctors and 39 nurses over 2 weeks, from November 3 to November 17, 2021. All respondents worked in the hospital both before and after implementation. The mobile questionnaires were filled out using Google.

### Outcome measures

The primary measure was ED LOS, and the secondary measure was the policy compliance rate. The time target policy requires 95% of patients to be admitted, discharged, or transferred from the ED within 24 hours of arrival. The policy compliance rate refers to the proportion of patients who successfully moved out from the ED within 24 hours. Along with ED LOS, the proportion of patients remaining in the ED after 24 hours was used as an indicator of the policy application.

Tertiary measures included the following outcomes: time to first prescription, time to admission decision, time to admission, time to computed tomography (CT), time to magnetic resonance imaging (MRI), time to operation, time to coronary angiography (CAG), and proportion of patient dispositions determined at 23 hours. All time variables, except time to admission, were calculated from the first presentation; time to admission was calculated from the time the decision to admit the patient was made.

### Survey

Patient flow, quality of care, patient safety, staff workload, need for improvement of the policy, and staff satisfaction levels were investigated through a questionnaire. Patient flow included the overall, triage, diagnostic evaluation and treatment, and disposition process. Quality of care was also assessed in terms of patient-centered, safe, effective, timely, efficient, and equitable treatment. In addition to the overall safety component in the quality of care section, questions for patient safety including patient identification, pressure ulcers, falls, medication, diagnostic tests, treatment, and others (infection-related, medical equipment, escape, violence, blood transfusion, etc.) were included. Workload dimensions, including mental, physical, and temporal demand, performance, effort, and degree of frustration, were also assessed. On top of the satisfaction of mefical staff with the policy, the degree to which medical staff felt the need for improvement in each patient flow was investigated. To develop a more comprehensive understanding of staff experiences, we included open-ended survey questions about aspects of the ED experience related to the time target policy (Supplementary table 1).

Questions about patient flow were written based on the input-throughput-output conceptual model of ED crowding suggested by Asplin et al. [4]. Questions about quality of care were based on the six domains of quality of care established by the Institute of Medicine and Medical Office Survey on Patient Safety Culture of the Agency for Healthcare Research and Quality. Questions on patient safety were based on the Korean Patient Safety Incident Report 2020 by the Korea Institute for Healthcare Accreditation [25, 26]. Questions about workload were based on the National Aeronautics and Space Administration Task Load Index [27].

### Statistical analysis

Continuous variables are presented as medians and interquartile ranges (IQRs) according to non-normal distributions on the Anderson-Darling test. Categorical variables are expressed as frequencies and percentages. To compare patient visits before and after policy implementation, the Mann–Whitney U test was used for continuous variables that were not normally distributed, and the χ2 test was used for categorical variables. *P*-values < .05 were considered statistically significant. All statistical analyses were performed using R (version 4.1.1; R Foundation for Statistical Computing, Vienna, Austria).

## Results

### Demographics

In total, 181,720 ED visits were examined across all sample periods. Patient demographics and clinical data before and after policy implementation are summarized in Table 1.

**Table 1:** Demographic and clinical characteristics of all ED patients

	Before policy implement (***N*** = 81,922)	After policy implement (***N*** = 99,798)	***P-***value
**Demographic**			
Age, years (IQR)	50 [25; 65]	52 [28; 66]	<.001
Sex			
Female	41,531 (50.7)	50,956 (51.1)	.124
**Clinical**			
Triage category: KTAS			<.001
1 (Resuscitation)	795 (1.0)	563 (0.6)	
2 (Emergency)	7101 (8.7)	5593 (5.6)	
3 (Urgent)	33,883 (41.4)	45,402 (45.5)	
4 (Semi-urgent)	32,115 (39.2)	40,987 (41.1)	
5 (Non-urgent)	6383 (7.8)	4966 (5.0)	
Missing	1645 (2.0)	2287 (2.3)	
Consciousness: AVPU			<.001
A (alert)	79,825 (97.4)	97,865 (98.1)	
V (response to verbal stimuli)	1099 (1.3)	865 (0.9)	
P (response to pain stimuli)	624 (0.8)	680 (0.7)	
U (unresponsive)	374 (0.5)	388 (0.4)	
Result			<.001
Discharge	56,639 (69.1)	70,786 (70.9)	
Death	308 (0.4)	350 (0.4)	
Admission	23,595 (28.8)	25,344 (25.4)	
Transfer	1380 (1.7)	3318 (3.3)	

*N* = 181,720. Age is presented as the median with interquartile range. Other categorical variables are presented as numbers (percentages, %). Comparison of patient visits before and after policy implementation used the Mann–Whitney U test for continuous variables that were not normally distributed and the χ2 test for categorical variables. Statistical significance was set at *p* < .05

*KTAS* Korean Triage and Acuity Scale

### Primary outcome: emergency department length of stay

**Fig. 1:**
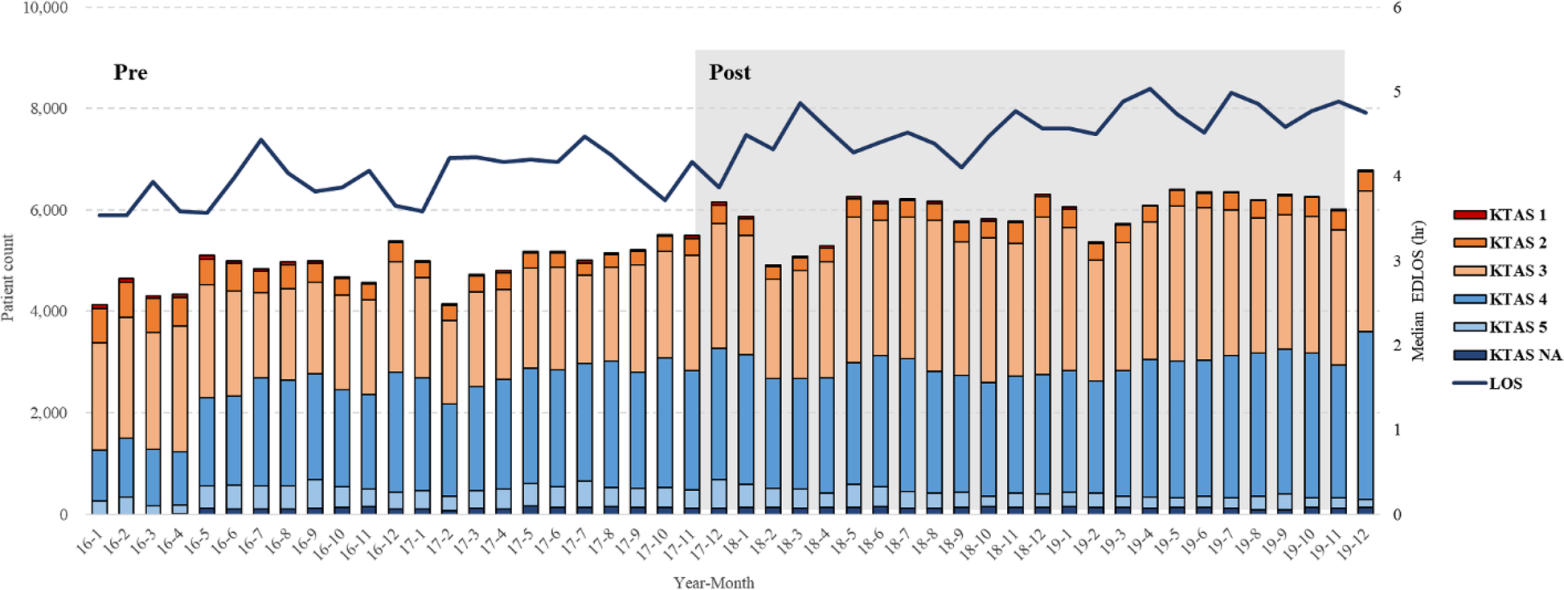
ED LOS and patient visits each month from February 1, 2016, to June 31, 2019. LOS is presented as a linear graph and patient visits as a bar. Proportion of KTAS level is indicated by bar color

Patient LOS in the ED during the study period is shown in Fig. 1. Compared with the period before policy implementation, median ED LOS increased from 3.9 hours (IQR = 2.1–7.6) to 4.5 hours (IQR = 2.5–8.5) as shown in Table 2. The average monthly number of patients before and after policy implementation was 4819 (*SD* = 340) and 5870 (*SD* = 462), respectively, a significant increase (*p* < .001). ED LOS and the number of patient visits are presented in Fig. 1.

**Table 2:** Primary outcome (ED LOS) and other outcomes related to time

	Before policy implementation	After policy implementation	***P***-value
	(***N*** = 81,922)	(***N*** = 99,798)	
ED LOS, hours	3.9 [2.1;7.6]	4.5 [2.5;8.5]	<.001
Time to first prescription, hours	0.4 [0.2; 0.6]	0.3 [0.2; 0.6]	<.001
Time to admission decision, hours^a^	4.3 [2.4; 7.8]	5.0 [2.7; 8.6]	<.001
Time to admission, hours^a^	2.1 [0.6; 7.8]	3.4 [1.2; 10.7]	<.001
Time to CT, hours^a^	1.7 [0.7; 3.1]	1.9 [0.7; 3.4]	<.001
Time to MRI, hours^a^	4.0 [2.6; 5.9]	3.9 [2.4; 5.9]	.001
Time to CAG, hours^a^	1.6 [0.9; 2.6]	2.4 [1.4; 3.9]	<.001
Time to operation, hours^a^	0.9 [0.3; 2.3]	0.8 [0.3; 2.3]	.755

All data are presented as medians with interquartile ranges. Comparison of ED LOS and other outcomes related to time before and after policy implementation used the Mann–Whitney U test for continuous variables that were not normally distributed. Statistical significance was set at *p* < .05

*ED LOS* Emergency department length of stay, *CT* Computed tomography, *MRI* Magnetic resonance imaging, *CAG* Coronary angiography

^a^The number of patients may differ from total number. Only those patients who underwent these tests and treatments were included in analysis

**Fig. 2:**
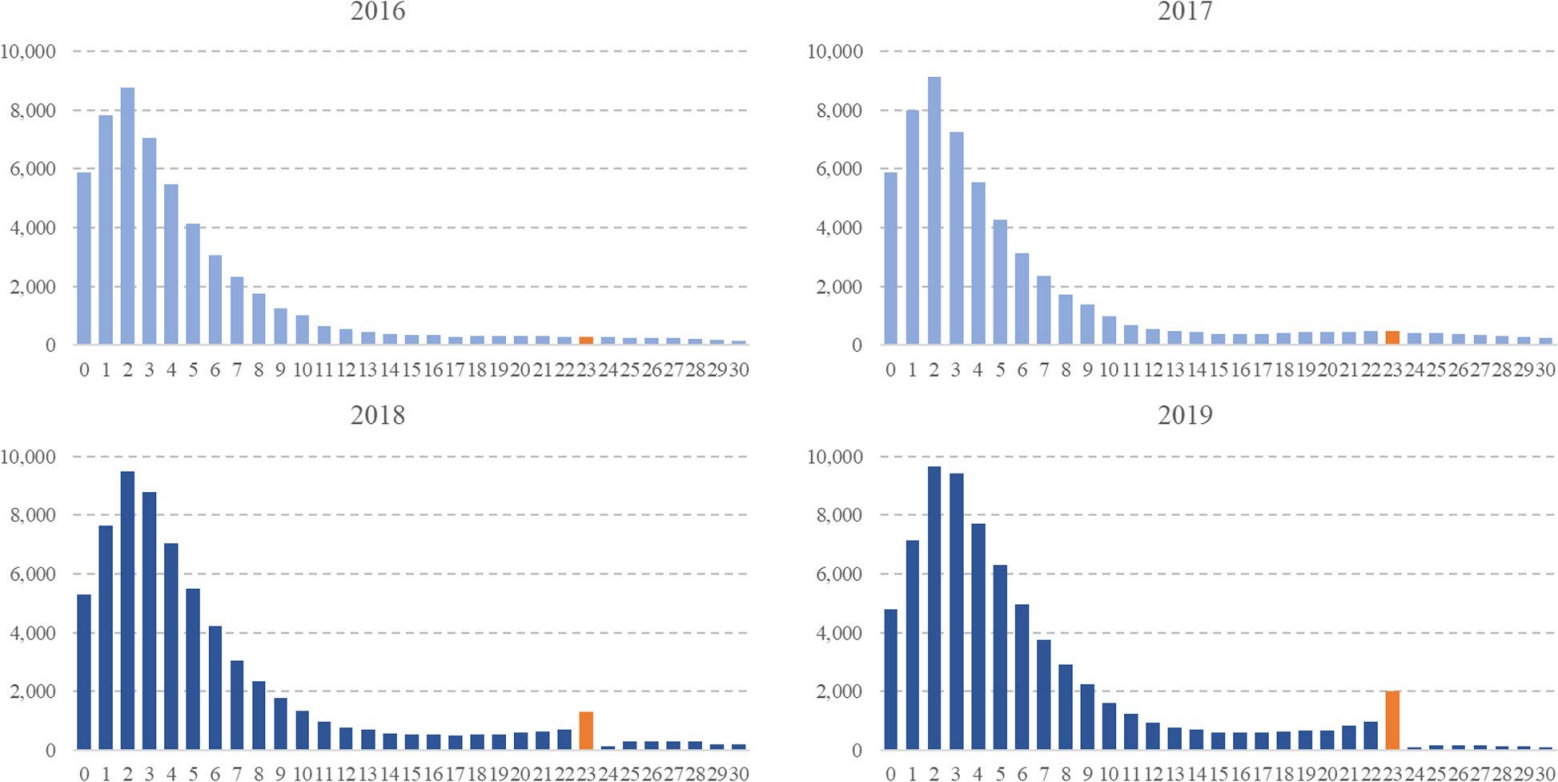
Hourly distribution of patients based on their emergency department length of stay. After policy introduced, event on 23 hour was shown in orange color

The hourly distribution of patients based on ED LOS is shown in Fig. 2. When comparing data collected before and after the policy, we found a significant increase in the number of patients whose disposition was decided at 23 hours, just before the target of 24 hours.

Supplementary table 2 shows a comparison between the pre- and post-policy periods by Korean Triage Acuity Scale (KTAS) group. Except for the KTAS 1 group, LOS increased after policy implementation.

### Secondary outcome: policy compliance rate

**Fig. 3:**
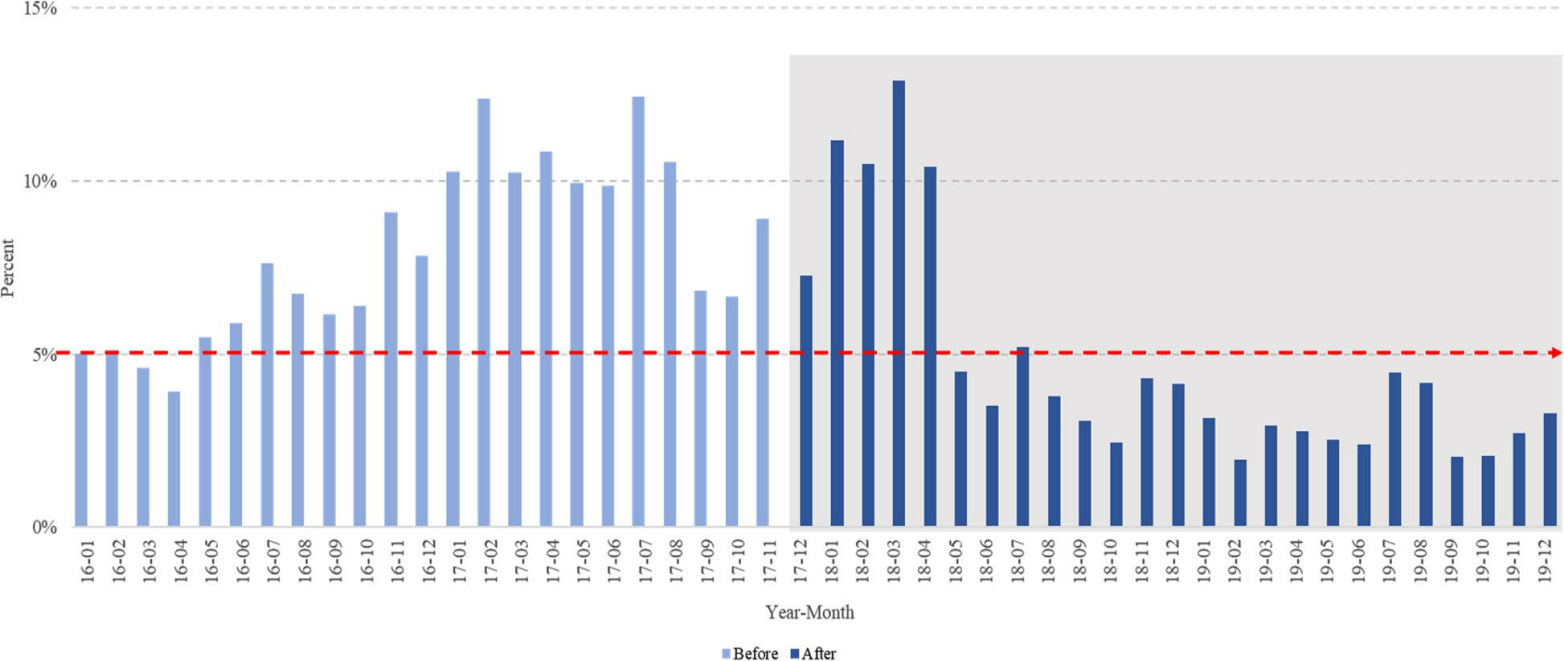
Proportion of patients who stayed for more than 24 hours from February 1, 2016, to June 31, 2019. The red dotted line represents 5% of total visit patients, which is the target proportion of the policy

As shown in Fig. 3, the proportion of patients who stayed for more than 24 hours before the policy was greater than 5% in most months. Even after the policy was introduced in December 2017, the proportion remained above 5%, but decreased dramatically after May 2018 and remained below 5% for the rest of the period.

### Tertiary outcomes

#### Time-related outcomes

Variables related to time are presented in Table 2, along with ED LOS. Time to the first prescription and time to MRI decreased slightly, but ED LOS, time to admission decision, time to admission, time to CT, and time to CAG increased significantly after the introduction of the policy. Patient distribution based on time to first prescription, admission decision-making, and CT and MRI procedures are presented in Supplementary fig. 1.

#### Disposition determined at 23 hours

The number of patients whose disposition was decided at 23 hours increased significantly after policy implementation (Fig. 2). Before the policy, the disposition of 698 patients was decided at 23 hours; however, after policy implementation, the number rose to 3384, an increase of almost 4.84-fold. The number of admitted patients whose disposition was determined at 23 hours increased significantly after the policy was implemented (Supplementary fig. 2).

### Questionnaire study outcomes

**Fig. 4:**
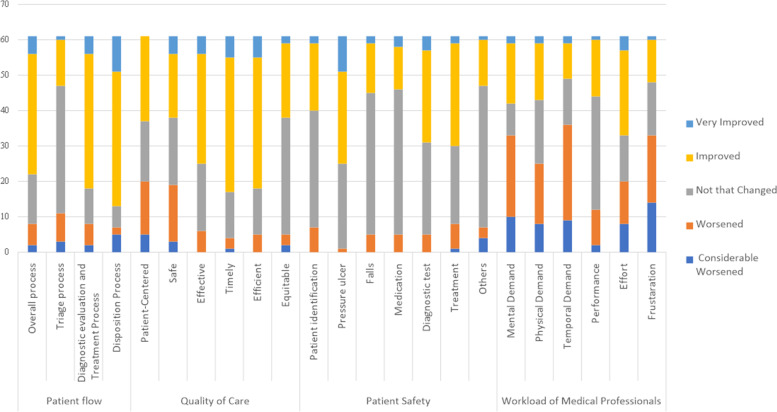
Survey response summary of ED medical professionals. Each color represents answer to each survey question. (Light blue: Very improved, Yellow: Improved, Gray: Not that changed, Orange: Worsened, Deep blue: Considerable worsened) Detailed data are presented in Supplementary table 4

To obtain in-depth information on changes in ED experiences before and after the introduction of 24-hour target policy, a survey was conducted among medical staff in the ED. A total of 61 medical staff members participated in the survey. Their demographics are presented in Supplementary table 3, and a summary of their responses is shown in Fig. 4, Supplementary table 4, Supplementary table 5 and Supplementary fig. 3.

With regard to patient flow, the most common response was that although the triage process did not change significantly, other processes improved (overall 55.7%, diagnostic evaluation and treatment 62.3%, and disposition 62.3%). Many respondents reported that quality of care also improved following introduction of the time target, particularly in terms of effectiveness (50.8%), timeliness (62.3%), and efficiency (60.7%). Regarding patient safety, some respondents reported improvements in pressure ulcers (42.6%) and treatment (47.5%). The survey also indicated that the overall workload of the medical professionals increased. Multiple responses indicated increasing mental (37.7%), physical (27.9%), and temporal demands (44.3%) along with rising levels of frustration (31.1%) (Supplementary table 4).

In a descriptive questionnaire, medical staff provided perspectives on their opposition to or approval of the time target policy with respect to patient flow, quality of care, patient safety, workload, need for improvement, and overall satisfaction (Supplementary tables 6, 7, 8, 9, 10 and 11).

The majority of medical staff respondents agreed that the decision-making process improved following the implementation of the time target policy, and almost half expressed satisfaction with the policy, but in a descriptive questionnaire, some noted decreased quality of information and treatment offered to patients due to time targets. Most medical staff pointed out that most ED processes, including triage, diagnosis and treatment, main department decision-making, and discharge, require further improvement (Supplementary tables 5, 6 and 7). Some respondents also described forceful discharges or transfers due to the time limitation, and some suggested that, to comply with the policy, not only the ED but the entire hospital system should work on improving ED flow (Supplementary tables 8, 10 and 11). However, all survey responses should be interpreted with caution, as they were obtained after the study period and may have been affected by recall bias.

## Discussion

To the best of our knowledge, this study is the first mixed-methods analysis of the impact of the 24-hour time target policy on ED experiences in Korea. The time target policy for EDs was introduced with the expectation of a whole-system approach to improving ED LOS [12]. This study found an increase in overall ED LOS and time to some ED processes despite good policy compliance.

In South Korea, patients can freely visit tertiary hospitals even when they don’t have a referral from a primary or secondary provider [28, 29]. This is unique among national healthcare systems, and the trend toward increasing demand at several already-crowded tertiary hospitals is intensifying because of the recent decision to strengthen health insurance coverage [30, 31]. Moreover, many South Korean patients are awaiting diagnosis and treatment for complicated chronic diseases or hospitalization for continued treatment following acute treatment in EDs [30]. The resulting crowding of EDs at tertiary hospitals led to the introduction of a 24-hour time target.

After implementing the time target policy, the proportion of patients with an LOS exceeding 24 hours decreased significantly, although the median ED LOS increased slightly (Figs. 1 and 3). Despite achieving the surface goal of reducing the proportion of patient stays exceeding 24 hours, the time target policy did not reduce LOS or improve the overall ED flow which were the policy’s ultimate goals. Even considering the possibility of patient severity differences between the two periods, Supplementary table 2 shows that LOS increased in all KTAS groups except KTAS 1. The increase in ED LOS can be attributed to a significant increase in the number of patient visits after policy implementation. Among the three flows described by Asplin [4], as input flow increases, more efforts is required to improve throughput and output flow. In the survey results, we observed similar complex responses; 63.9% of medical staff reported that patient overall flow seems to be improved or very improved (Supplementary table 4), and 47% of respondents were satisfied with the policy while 36% were not.

In this study, ED LOS distribution for 23 hours, just before the time target, changed before and after the policy. This finding is consistent with earlier research that found that, when a time target policy was implemented in other nations, including the UK, Australia, and New Zealand, an ED patient’s disposition tended to be determined just before the time target was reached [24, 32, 33]. As a result, it is unclear whether the decrease in the proportion of patients staying in the ED for more than 24 hours was due to improved patient flow or to “gaming” the system by seeming to comply with the policy, as suggested by Tenbensel et al [24]. Some survey participants in this study also described such “gaming” practices, along with the forceful transfer and discharge of patients. They also noted that urgent transfers or discharges could threaten patient safety owing to treatment discontinuity and insufficient medical staff in wards.

Despite the policy implementation, the time to critical tests and interventions which are classified as the throughput flow of Asplin [4], increased further. The change in patient distribution clearly demonstrated an increase in the number of patients who needed tests and interventions during the same period (Supplementary fig. 1). Therefore, given the increased time to individual examinations and interventions, the distribution of ED LOS of 23 hours, and “game” effect, only the superficial goal of a 5%, the proportion of patients who stayed more than 24 hours, was achieved and it is likely that the time target policy didn’t work for improving LOS or the overall ED flow. It may be difficult to achieve the policy’s goal without controlling the input flow in Korea’s national health system, where health insurance coverage continues to increase and patients have a wide range of hospital options and a strong preference for tertiary referral hospitals.

A previous study in New Zealand showed ED LOS monitoring strategies including the display of real-time information for ED LOS and the operation of short-term emergency wards that only admit patients from the ED can help lower ED LOS and improve patient flow [24]. Considering that the policy compliance rate was improved in the several months following the introduction of the time target policy, it can be expected that additional efforts were made to improve throughput and output flow. The operation of emergency wards that admit patients only from the ED, a dedicated transfer-coordinator nurse system for EDs, LOS management implemented by each department, and LOS monitoring within the ED would have been helpful in effectively managing policy compliance rate in target hospitals [34–37]. As such, if it is difficult to improve the input flow in the Korean healthcare system, looking at other throughput and output flows can be an alternative.

Meanwhile, unless there is a change in other conditions such as the number of medical staff, the fact that the timing of first prescriptions and tests are similar with the  increased number of patient visits suggests that the burden on medical staff might have increased. The survey also identified increased workload among medical professionals. Medical staff reported that patient flow, quality of care, and some patient safety indicators were improved by the policy, but the workload of the medical professionals was greater than before the policy. Overall, the higher compliance rate despite the increased input flow represents the increased workloads of medical staff, which was presented in the survey. To pursue the target, the pressure to discharge a patient within 24 hours may also influence staff workload. Whether it is a “gaming” effect or an improvement in patient flow, the fact that the disposition of many patients is hastily decided at the 23rd hour compared to before the policy seems to have caused additional workload for the medical staff (Fig. 2).

The downside of this policy is that the medical conditions of patients are not considered. Patients who require additional workup or emergency care can be admitted to the ward or transferred to comply with the policy. Patients who require hospitalization often require more treatment time than patients who return home, as noted in previous studies [32, 38]. In Canada, the target time varies depending on the severity of the disease or trauma and the acuity level of the triage stage; this approach could be applied in South Korea [14]. In our survey, medical staff also expressed concerns about unified policy applications. Some medical personnel noted due to the pressure to comply with the time target, time and opportunities to take care of patients are insufficient.

Our findings suggest several approaches for improving the implementation of the time target policy. First, such policies require support from outside the ED, including an increased ward capacity and improved transfer systems. Second, each flow of ED, including triage, prescription, lab test, imaging tests, admission or discharge should be monitored and backed up to improve the entire ED process. Third, the workload of medical staff should be considered, and appropriate compensation should be offered when policy compliance is high. Fourth, disease severity of patients should be considered. Patients who visit tertiary hospitals have relatively high disease severity, and in many cases, it is difficult to transfer them to other hospitals. For patient safety, it may be helpful to adjust the target time according to patients’ severity, such as KTAS level or disposition, rather than applying a unified time target to all patients. Fifth, policies should be tailored based on the characteristics of each hospital. Each hospital has different patient characteristics and resources. In Korea, the severity and number of patients vary according to the ED location. Usually, patients with ED in metropolitan areas have higher severity and larger numbers. In tertiary referral hospitals located in metropolitan areas, patients often come from other tertiary general hospitals to receive appropriate treatment and additional medical resources. Policymakers should consider these factors to improve patient flow in Korea.

## Limitations

This study has some limitations. As this was a single-center study, we were unable to exclude selection bias, and our results might not be generalizable. However, various participants and circumstances can be recruited from the study site, which is an crowded tertiary referral hospital located in an urban area. This study attempted to show several aspects of the implementation of the target policy such as the ED LOS, the time change of each process, and the ED LOS distribution. In addition, a survey was conducted on several aspects, including opinions on patient flow, quality care, patient safety, workload, and the process that needs improvement. Hospitals in similar environments can obtain a rich perspective from this study. Second, retrospective surveys face the risk of recall bias. An in-depth interview study might be required to improve quality assessment. Thirdly, the large study population must be considered when assessing the study’s results and the *p*-value [39]. The interquartile range and sample size were all expressed for additional interpretation. In addition, the time difference before and after the policy shownin Table 2can be clinically meaningful, even if it only 0.5 hours, and can influence all of LOS, overall ED process, and patient outcomes. Fourthly, Due to potential confounding factors, the interrupted time series analysis was not performed. Trends are susceptible to change at various times, especially during certain seasons. Instead, the research period was divided into three parts: pre-policy (February 2016 to June 2017), adjustment period (July 2017 to January 2018), and post-policy (February 2018 to June 2019), with concurrent months separating the pre-policy and post-policy groups. Finally, during the study period, other actions to improve ED flow were implemented or were already in place that could influence the ED LOS. The Ministry of Health and Welfare conducts annual quality evaluation through indicators such as the ED LOS of patients with severe ill code and the proportion of severe patients with high KTAS levels who were directly evaluated in a timely manner by emergency medicine specialists [18, 40]. This was another reason that interrupted time series analysis was hard to perform. It was impossible to consider all these factors simultaneously because of the retrospective observational nature of the study. Instead, we focused on analyzing the Korean 24-hour target policy from a variety of perspectives, including both empirical data and surveys.

## Conclusions

After South Korea implemented a 24-hour time target policy for EDs, the proportion of patients whose LOS exceeded 24 hours decreased, although the median ED LOS increased slightly. However, unintended consequences of the policy were also observed such as increased medical staff workload and abrupt expulsion of patients before 24 hours.

## Supplementary Information

**Additional file 1: Supplementary Table 1.** Survey. **Supplementary Table 2.** Comparison emergency department length of stay between before and after policy by each KTAS group. **Supplementary Fig. 1.** Patient distribution: time spent on first prescription, admission decision, computed tomography, and magnetic resonance imaging. **Supplementary Fig. 2.** The number of patients whose disposition was decided at 23 hours. **Supplementary Table 3.** Demographic data for survey participants. **Supplementary Table 4.** Anwsers to survey part A-D. “Evaluation of changes in ED after implementing the policy”. **Supplementary Table 5.** Answers to survey part E. “Need for improvement in the policy”. **Supplementary Fig. 3.** Answers to survey part F. “Satisfaction with the policy”. **Supplementary Table 6.** Answers to question 5. “Please feel free to write your opinion on the patient flow of the emergency department after implementing the 24-hour time target policy”. **Supplementary Table 7.** Answers to question 12. “Please feel free to write your opinion on the quality of care of the emergency department after implementing the 24-hour time target policy”. **Supplementary Table 8.** Answers to question 20. “Please feel free to write your opinion on the patient safety of the emergency department after implementing the 24-hour time target policy”. **Supplementary Table 9.** Answers to question 27. “Please feel free to write your opinion on the workload of the emergency department after implementing the 24-hour time target policy”. **Supplementary Table 10.** Answers to question 32. “Please feel free to write your opinion about the process that needs improvement to reduce the length of stay in the emergency department”. **Supplementary Table 11.** Answers to question 34. “Please feel free to write your opinion on the overall satisfaction of the emergency department after implementing the 24-hour time target policy”.

## Data Availability

Data are available from the clinical data warehouse of the study site. The datasets generated and analyzed during the current study are not publicly available, but are available from the corresponding author upon reasonable request.
