# Supplementary material for: Impact of the 24-hour time target policy for emergency departments in South Korea: a mixed method study in a single medical center
Source: BMC Health Serv Res. 2022 Dec 12;22:1510. doi: 10.1186/s12913-022-08861-y (PMC9742653; doi:10.1186/s12913-022-08861-y)
Supplement: Supplementary file 1 — Additional file 1: Supplementary Table 1. Survey. Supplementary Table 2. Comparison emergency department length of stay between before and after policy by each KTAS group. Supplementary Fig. 1. Patient distribution: time spent on first prescription, admission decision, computed tomography, and magnetic resonance imaging. Supplementary Fig. 2. The number of patients whose disposition was decided at 23 hours. Supplementary Table 3. Demographic data for survey participants. Supplementary Table 4. Anwsers to survey part A-D. “Evaluation of changes in ED after implementing the policy”. Supplementary Table 5. Answers to survey part E. “Need for improvement in the policy”. Supplementary Fig. 3. Answers to survey part F. “Satisfaction with the policy”. Supplementary Table 6. Answers to question 5. “Please feel free to write your opinion on the patient flow of the emergency department after implementing the 24-hour time target policy”. Supplementary Table 7. Answers to question 12. “Please feel free to write your opinion on the quality of care of the emergency department after implementing the 24-hour time target policy”. Supplementary Table 8. Answers to question 20. “Please feel free to write your opinion on the patient safety of the emergency department after implementing the 24-hour time target policy”. Supplementary Table 9. Answers to question 27. “Please feel free to write your opinion on the workload of the emergency department after implementing the 24-hour time target policy”. Supplementary Table 10. Answers to question 32. “Please feel free to write your opinion about the process that needs improvement to reduce the length of stay in the emergency department”. Supplementary Table 11. Answers to question 34. “Please feel free to write your opinion on the overall satisfaction of the emergency department after implementing the 24-hour time target policy”. [file 12913_2022_8861_MOESM1_ESM.pdf]

Supplementary Table 1. Survey

A. Patient flow

Please indicate your evaluation of changes in the emergency department after implementing the 24-hour time target policy for each sentence.

1. Overall process	Considerable worsened	Worsened	Not that changed	Improved	Very improved
2. Triage process	Considerable worsened	Worsened	Not that changed	Improved	Very improved
3. Diagnostic evaluation and treatment process	Considerable worsened	Worsened	Not that changed	Improved	Very improved
4. Disposition process	Considerable worsened	Worsened	Not that changed	Improved	Very improved

5. Please feel free to write your opinion of the patient flow of the emergency department after implementing the 24-hour time target policy.

B. Quality of Care

Please indicate your evaluation of changes in the emergency department after implementing the 24-hour time target policy for each sentence.

6. Patient-centered<br>Is responsive to individual patient preferences, needs, and values	Considerable worsened	Worsened	Not that changed	Improved	Very improved
7. Safe<br>Prevents harm from treatment to help patients.	Considerable worsened	Worsened	Not that changed	Improved	Very improved
8. Effective<br>Is based on scientific knowledge	Considerable worsened	Worsened	Not that changed	Improved	Very improved
9. Timely<br>Minimizes waits and potentially harmful delays	Considerable worsened	Worsened	Not that changed	Improved	Very improved
10. Efficient<br>Ensures cost-effective care (avoids waste, overuse, and misuse of services)	Considerable worsened	Worsened	Not that changed	Improved	Very improved

11. Equitable<br>Provides the same quality of care to all individuals regardless of gender, race, ethnicity, socioeconomic status, language, etc.	Considerable worsened	Worsened	Not that changed	Improved	Very improved

12. Please feel free to write your opinion of the quality of care of the emergency department after implementing the 24-hour time target policy.

____

C. Patient Safety

Please indicate your evaluation of changes in the emergency department after implementing the 24-hour time target policy for each sentence.

13. Patient identification	Considerable worsened	Worsened	Not that changed	Improved	Very improved
14. Pressure ulcer	Considerable worsened	Worsened	Not that changed	Improved	Very improved
15. Falls	Considerable worsened	Worsened	Not that changed	Improved	Very improved
16. Medication	Considerable worsened	Worsened	Not that changed	Improved	Very improved
17. Diagnostic test	Considerable worsened	Worsened	Not that changed	Improved	Very improved
18. Treatment	Considerable worsened	Worsened	Not that changed	Improved	Very improved
19. Others (infection-related, medical equipment, escape, violence, blood transfusion, etc.)	Considerable worsened	Worsened	Not that changed	Improved	Very improved

20. Please feel free to write your opinion of the patient safety of the emergency department after implementing the 24-hour time target policy.

____

D. Workload

Please indicate your evaluation of changes in the emergency department after implementing the 24-hour time target policy for each sentence.

21. Mental demand<br>- How mentally demanding was the task?	Considerable worsened	Worsened	Not that changed	Improved	Very improved
22. Physical demand<br>- How physically demanding was the task?	Considerable worsened	Worsened	Not that changed	Improved	Very improved
23. Temporal demand<br>- How hurried or rushed was the pace of the task?	Considerable worsened	Worsened	Not that changed	Improved	Very improved
24. Performance<br>- How successful were you in accomplishing what you were asked to do?	Considerable worsened	Worsened	Not that changed	Improved	Very improved
25. Effort<br>- How hard did you have to work to accomplish your level of performance?	Considerable worsened	Worsened	Not that changed	Improved	Very improved
26. Frustration<br>- How insecure, discouraged, irritated, stressed, and annoyed were you?	Considerable worsened	Worsened	Not that changed	Improved	Very improved

27. Please feel free to write your opinion of the workload of the emergency department after implementing the 24-hour time target policy.

E. Need for improvement of the policy

Please indicate your assessment of the additional need for improvement to reduce the length of stay in the emergency department.

28. Triage	Strongly disagree	Disagree	Neither agree nor disagree	Agree	Strongly Agree
29. Diagnosis and treatment	Strongly disagree	Disagree	Neither agree nor disagree	Agree	Strongly Agree
30. Decision of the main department	Strongly disagree	Disagree	Neither agree nor disagree	Agree	Strongly Agree
31. Discharge	Strongly disagree	Disagree	Neither agree nor disagree	Agree	Strongly Agree

32. Please feel free to write your opinion of the process that needs improvement to reduce the length of stay in the emergency department.

F. Satisfaction with the policy

Please indicate your evaluation of satisfaction with the 24-hour time target policy.

33. Overall satisfaction	Very unsatisfactory	Unsatisfactory	Neither satisfactory nor unsatisfactory	Satisfactory	Very satisfactory

34. Please feel free to write your opinion about the satisfaction with the 24-hour time target policy

---

Supplementary Table 2. Comparison emergency department length of stay between before and after policy by each KTAS group

	Before policy implementation (N = 81,922)	After policy implementation (N = 99,798)	*P*-value
Triage category: KTAS			
1 (Resuscitation)	4.15	2.40	<.001
2 (Emergency)	5.03	6.12	<.001
3 (Urgent)	5.00	5.80	<.001
4 (Semi-urgent)	3.18	3.63	<.001
5 (Non-urgent)	2.47	2.55	<.001
Missing	1.62	1.52	.201

Note. N=181,720. All data are presented as hours. The comparison of length of stay between pre- and post-policy implementation applied the Mann–Whitney U test for continuous variables that were not normally distributed. P-values < 0.05 were considered statistically significant.
KTAS: Korean Triage and Acuity Scale

Except KTAS 1 group, LOS had increased in after policy implementation.

Supplementary Fig 1. Patient distribution: time spent on first prescription, admission decision, computed tomography, and magnetic resonance imaging.

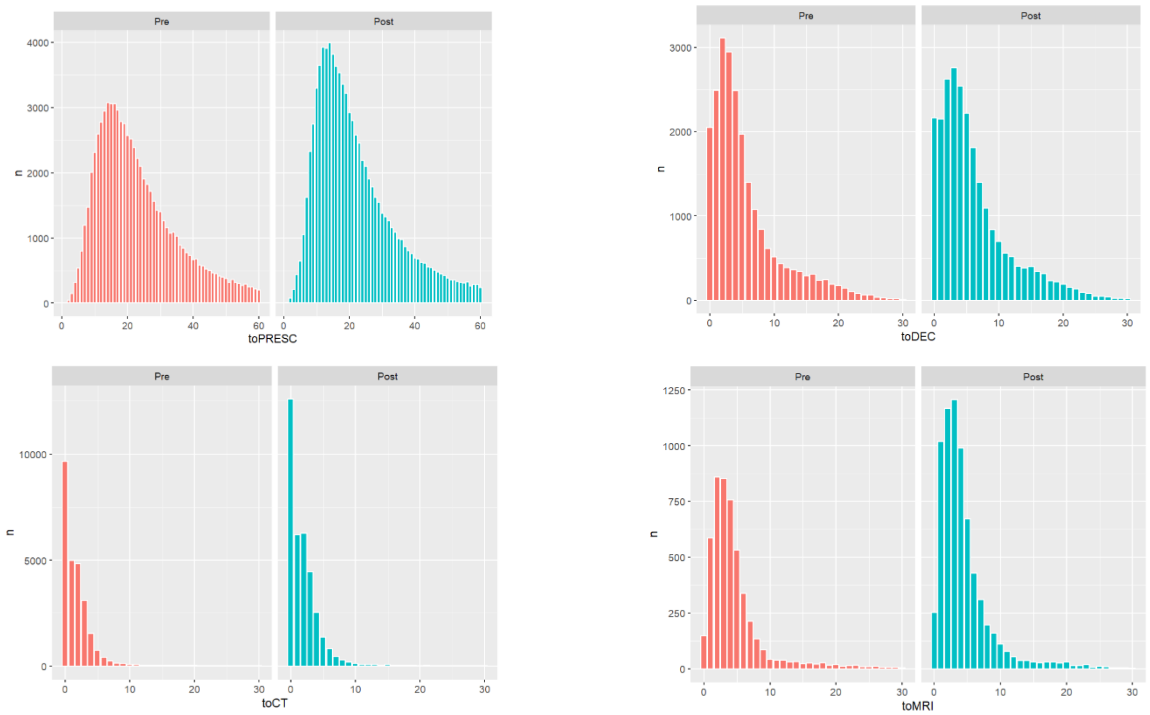

Figure shows time spent on each clinical action. toPRESC: to first prescription, toDEC: to admission decision, toCT: to computed tomography (CT), toMRI: to magnetic resonance imaging. In the case of time to the first prescription, it was described on basis of minute units, and the other three were described on basis of hour units.

Supplementary Fig 2. The number of patients whose disposition was decided at 23 hours

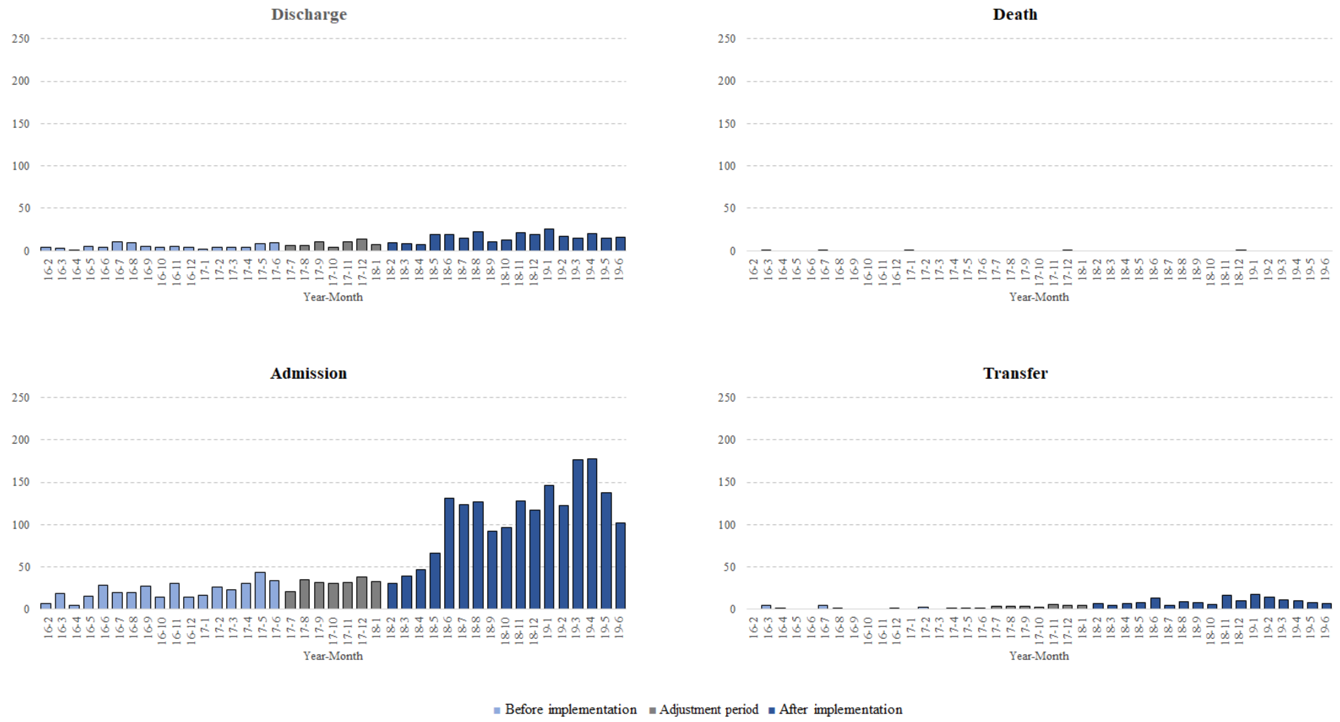

Figures show patient discharge, admission, death, and transfer rates at 23 hours by month.

Supplementary Table 3. Demographic data for survey participants

	Doctor (N=22)	Nurse (N=39)
Demographic		
Age (years)	35.5 ± 3.1	32 ± 4.4
Sex		
Female	10 (45.5%)	33 (84.6%)
Male	12 (54.5%)	6 (15.4%)
Department		
Emergency	16 (72.7%)	36 (92.3%)
Others	6 (27.3%)	3 (7.7%)
Working years	8.2 ± 3.9	5.8 ± 2.9

Note. N=61. Age and working years were reported as means and standard deviations, SDs.

Other data were reported as numbers (percentages, %).

Supplementary Table 4. Anwsers to survey part A-D. "Evaluation of changes in ED after implementing the policy"

	Considerable Worsened	Worsened	Not that Changed	Improved	Very improved
Patient Flow					
Overall process	2 (3.3)	6 (9.8)	14 (23.0)	34 (55.7)	5 (8.2)
Triage process	3 (4.9)	8 (13.1)	36 (59.0)	13 (21.3)	1 (1.6)
Diagnostic evaluation and treatment process	2 (3.3)	6 (9.8)	10 (16.4)	38 (62.3)	5 (8.2)
Disposition process	5 (8.2)	2 (3.3)	6 (9.8)	38 (62.3)	10 (16.4)
Quality of Care					
Patient-centered	5 (8.2)	15 (24.6)	17 (27.9)	24 (39.3)	0 (0.0)
Safe	3 (4.9)	16 (26.2)	19 (31.1)	18 (29.5)	5 (8.2)
Effective	0 (0.0)	6 (9.8)	19 (31.1)	31 (50.8)	5 (8.2)
Timely	1 (1.6)	3 (4.9)	13 (21.3)	38 (62.3)	6 (9.8)
Efficient	0 (0.0)	5 (8.2)	13 (21.3)	37 (60.7)	6 (9.8)
Equitable	2 (3.3)	3 (4.9)	33 (54.1)	21 (34.4)	2 (3.3)
Patient Safety					
Patient identification	0 (0.0)	7 (11.5)	33 (54.1)	19 (31.1)	2 (3.3)
Pressure ulcer	0 (0.0)	1 (1.6)	24 (39.3)	26 (42.6)	10 (16.4)
Falls	0 (0.0)	5 (8.2)	40 (65.6)	14 (23.0)	2 (3.3)
Medication	0 (0.0)	5 (8.2)	41 (67.2)	12 (19.7)	3 (4.9)
Diagnostic test	0 (0.0)	5 (8.2)	26 (42.6)	26 (42.6)	4 (6.6)
Treatment	1 (1.6)	7 (11.5)	22 (36.1)	29 (47.5)	2 (3.3)
Others	4 (6.6)	3 (4.9)	40 (65.6)	13 (21.3)	1 (1.6)
Workload of Medical Professionals					
Mental demand	10 (16.4)	23 (37.7)	9 (14.8)	17 (27.9)	2 (3.3)
Physical demand	8 (13.1)	17 (27.9)	18 (29.5)	16 (26.2)	2 (3.3)
Temporal demand	9 (14.8)	27 (44.3)	13 (21.3)	10 (16.4)	2 (3.3)
Performance	2 (3.3)	10 (16.4)	32 (52.5)	16 (26.2)	1 (1.6)
Effort	8 (13.1)	12 (19.7)	13 (21.3)	24 (39.3)	4 (6.6)
Frustration	14 (23.0)	19 (31.1)	15 (24.6)	12 (19.7)	1 (1.6)

Note. N=61. Data were reported as numbers (percentages, %)

Supplementary Table 5. Answers to survey part E. "Need for improvement in the policy"

	Triage	Diagnosis and treatment	Decision of the main department	Discharge
Strongly agree	17 (28)	16 (26)	39 (64)	25 (41)
Agree	28 (46)	33 (54)	18 (30)	23 (38)
Neither Agree nor Disagree	9 (15)	10 (16)	4 (7)	7 (11)
Disagree	7 (11)	2 (3)	0 (0)	6 (10)
Strongly disagree	0 (0)	0 (0)	0 (0)	0 (0)

Note. N=61. Data were reported as numbers (percentages, %)

Supplementary Fig 3. Answers to survey part F. “Satisfaction with the policy”

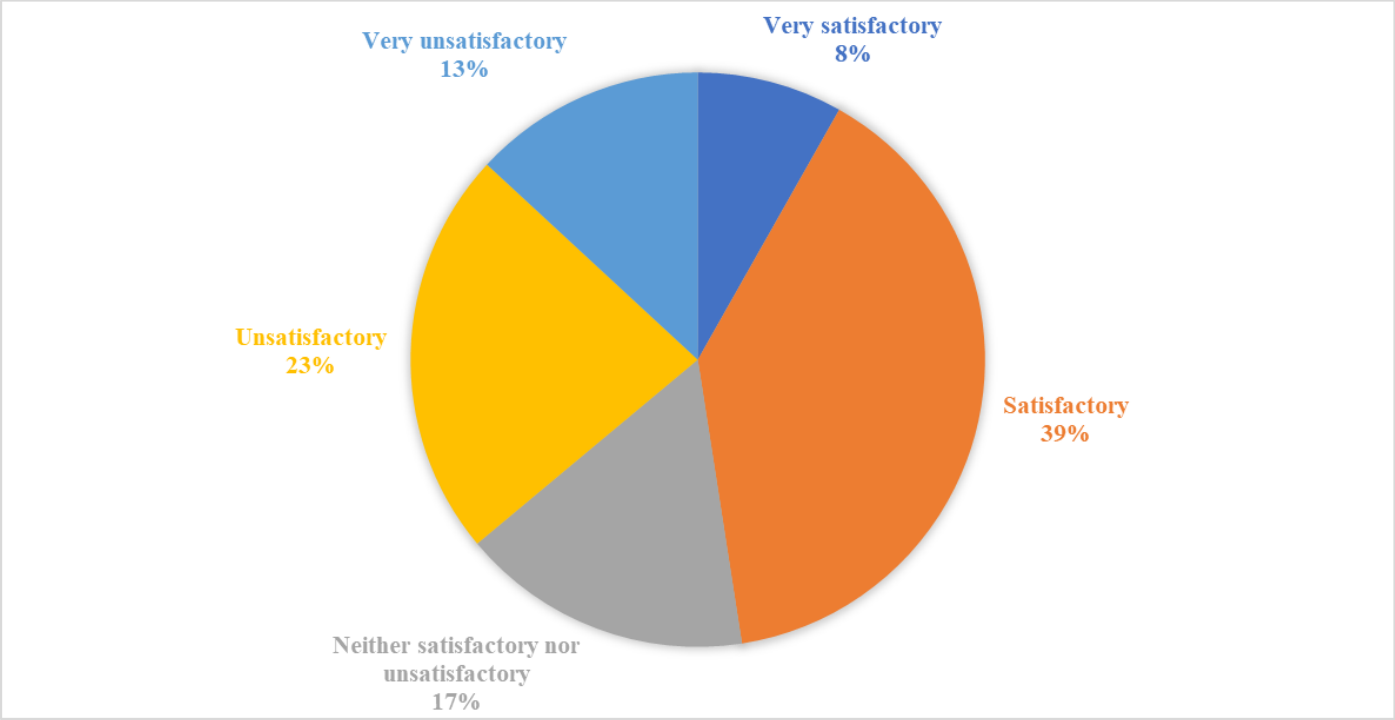

Figure shows overall satisfaction of 61 medical professionals with the 24-hour target policy.

Supplementary Table 6. Answers to question 5. "Please feel free to write your opinion on the patient flow of the emergency department after implementing the 24-hour time target policy."

	Answer
Pros	Length of stay of patient seems to be decreased<br>Decision making process become faster<br>Easy to explain to patient and other departments about need of fast decision-making in ED process
Cons	Decreased quality of explanation or consultation about treatment flow because of a lack of time<br>As the policy only applies in the ED, delay of admission or other problems of flow of another parts of hospital system are not solved

Supplementary Table 7. Answers to question 12. “Please feel free to write your opinion on the quality of care of the emergency department after implementing the 24-hour time target policy.”

	Answer
Pros	Faster decision making due to time limitation
	Faster outflow of patients and faster inflow
Cons	Insufficient exam and care for patient to hasten the process
	Increased revisit rate due to insufficient care
	Insufficient explanation to patient due to lack of time
	Unreasonable and forceful transfer/discharge
	Focusing on decision making and treatment to achieve the time goal

Supplementary Table 8. Answers to question 20. “Please feel free to write your opinion on the patient safety of the emergency department after implementing the 24-hour time target policy.”

	Answer
Pros	Decreased fall due to faster admission<br>Improved wound care because of faster decision making and decreased time stay in ED
Cons	Increased transfer or admission at night-time due to insufficient medical staff, which can threaten patient safety<br>Forceful discharge or transfer increases<br>Urgent transfer or discharge at the last hour could threaten patient safety because of discontinuity of treatment

Supplementary Table 9. Answers to question 27. "Please feel free to write your opinion on the workload of the emergency department after implementing the 24-hour time target policy."

	Answer
Pros	Easy to share opinions for fast decisions because of regulation<br>Easy to explain to the patient about transfer or discharge<br>Easy to cooperate with support department due to policy
Cons	The workload for transfer increased<br>Time limitation itself can be a workload, particularly for departments directly related to the emergency department<br>Workload increases due to patient complaints about forcing transfer or discharge<br>Workload increases due to increased turnover of patients

Supplementary Table 10. Answers to question 32. "Please feel free to write your opinion about the process that needs improvement to reduce the length of stay in the emergency department"

Answer
All processes need improvement
Need to improve admission process
Need to improve contact and cooperation with other departments
Need to increase threshold to enter emergency department
Non-emergent procedure should be performed after admission

Supplementary Table 11. Answers to question 34. "Please feel free to write your opinion on the overall satisfaction of the emergency department after implementing the 24-hour time target policy."

Answer
It is not easy to enforce the policy, especially for patients
The policy does not consider the characteristics of the patient's individual disease consideration or preparation for procedure or surgery
Need more cooperation from other departments outside of the ED
More education for patient guardians and other support
Need to avoid transfer from outpatient clinics to the emergency department for admission due to lack of ward depletion.
Avoid transfers between hospitals that are not communicated, as this can result in an inflow overload in an incapable emergency room, which can lead to increased length of stay due to increased waiting time. It should be subordinate to other policies.
